# Risk Prediction for Heart Failure in Patients with Cancer: Current Models and Future Directions

**DOI:** 10.1007/s11897-026-00793-6

**Published:** 2026-11-10

**Authors:** Arianne Clare Agdamag, Alexandra S. Manaila-Nabi, Phoo Pwint Nandar, W. H. Wilson Tang

**Affiliations:** 1 Cleveland Clinic, Cleveland, OH United States; 2 Metrohealth, Cleveland, OH United States

**Keywords:** Cardio-oncology, Heart failure, Risk prediction, Prediction models

## Abstract

Purpose of Review: Cancer survivors have an elevated risk for developing heart failure due to prior chemotherapy, radiation therapy, and other overlapping co-morbid conditions. With the advancement in cancer treatment, there is an increasing number of survivors who are at an even higher risk of developing heart failure. There is a growing need for heart failure specific risk prediction models in this population to facilitate early diagnosis and timely management. This review aims to summarize most recent findings on heart failure risk prediction among patients with history of cancer. Recent findings: Current risk prediction models focus on heart failure risk at the time of cancer treatment and shortly thereafter. There is limited data on heart failure risk among long term survivors.

**Graphical Abstract:**

Central Figure: Approach in determining heart failure risk before, during, and, after cancer treatment

**Figure:**
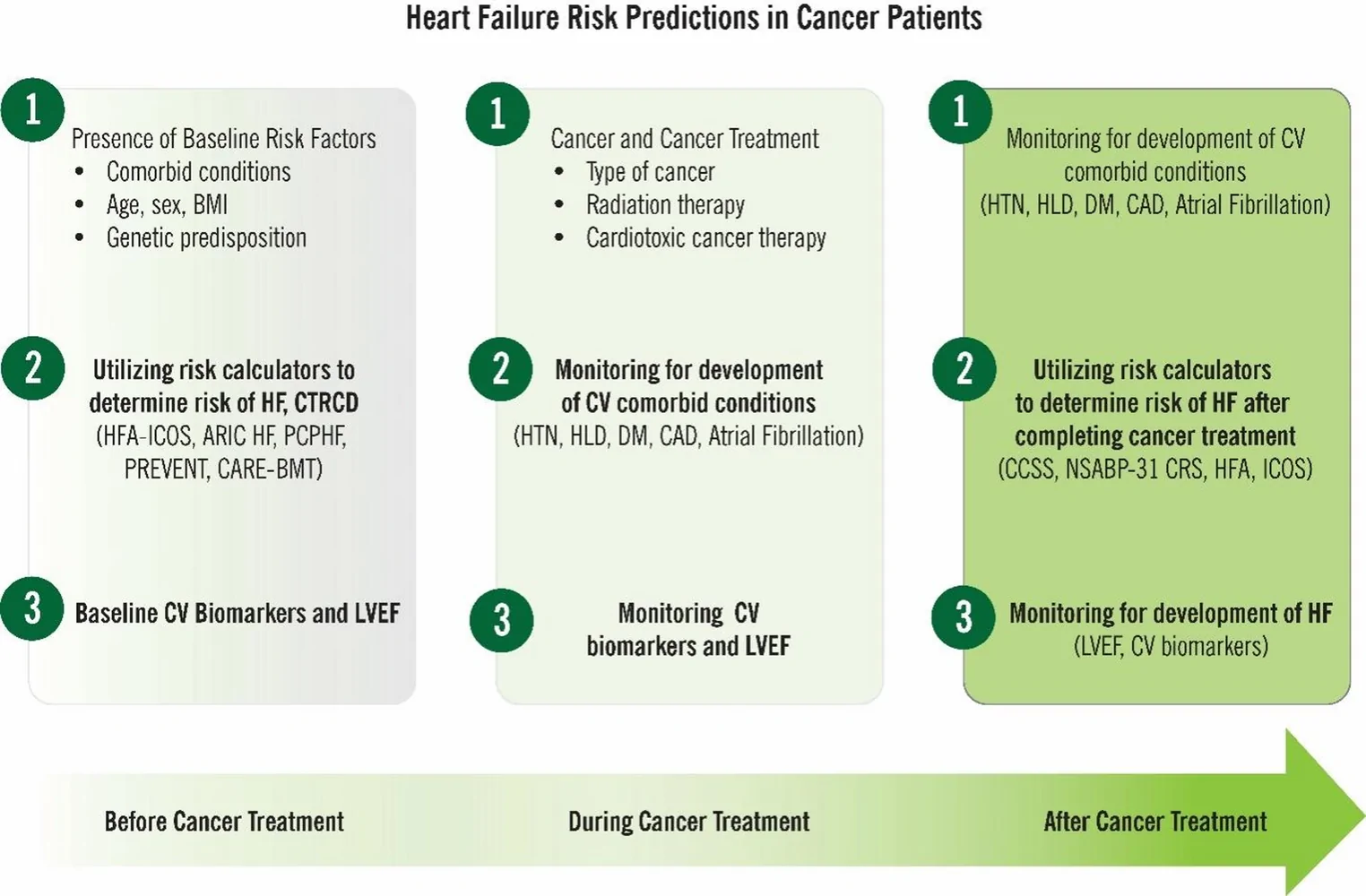

## Introduction

Cancer and cardiovascular disease are two leading causes of morbidity and mortality. Their intersection has become a rapidly evolving area of clinical and scientific inquiry [1]. Cancer survivors are at increased risk of developing cardiovascular disease, especially heart failure [2]. This is attributed to overlapping risk factors such as hyperlipidemia, hypertension, diabetes, obesity, and smoking that are associated with the development of cancer and cardiovascular disease. However, the overlap between these conditions extends beyond traditional risk factors and is now understood to involve shared genetic, molecular, and biological substrates [1, 3, 4]. 

At the genetic level, clonal hematopoiesis of indeterminate potential (CHIP) has emerged as a key link between cancer and heart failure [5]. CHIP, defined by the clonal expansion of hematopoietic stem cells harboring somatic mutations in certain genes, is present in approximately 10% of individuals over 70 years of age [6]. While CHIP is recognized as a precursor to hematologic malignancies with a 0.5–1.0% annual risk of progression, it simultaneously confers a nearly two-fold increase in cardiovascular risk compared to those without CHIP.[6, 7].

In addition to these shared predispositions, cancer therapies themselves are a well-established cause of cardiac injury. Anthracyclines, HER2-targeted agents, immune checkpoint inhibitors, and thoracic radiation can each cause direct myocardial damage leading to heart failure. More recently, however, a growing body of evidence suggests that the relationship between cancer and heart failure may not be unidirectional. Instead, heart failure itself may also promote tumor development [8].

The recognition that heart failure and cancer are not independent entities but rather interconnected conditions with shared epidemiology and biology underscores the urgent need for robust, validated risk predication tools tailored to this population. As survival improves among cancer patients, it is important to identify which patients are at high risk of developing heart failure as early identification leads to timely treatment and reduction of health care cost [9]. This review article aims to discuss chemotherapeutic and immunotherapy agents and their risk of heart failure, the incidence of heart failure among cancer survivors, and the existing risk calculators in predicting heart failure among cancer patients.

## Epidemiology of Cancer-Related Heart Failure

Heart failure among cancer patients can be a pre-existing condition, can occur during cancer treatment, and after completion of cancer therapy. The actual incidence of heart failure during these different time points remains unclear as most data has focused on investigating cancer therapy related cardiac dysfunction (CTRCD) with limited studies investigating heart failure with preserved ejection fraction. In a large population study, approximately 3% of patients with newly diagnosed cancer were noted to have heart failure [10]. The risk of developing heart failure during cancer treatment depends on baseline risk factors, type of cancer, and type of therapy. The risk of heart failure among adult and childhood cancer survivors is further discussed below. Notably, the main risk factors for heart failure development differ between these two populations: in adults, aging and comorbid conditions such as hypertension, diabetes, and coronary artery disease are the primary contributors, whereas in childhood cancer survivors, the cumulative exposure to cardiotoxic therapies — particularly anthracyclines and chest-directed radiation — plays a more central role.

### Heart Failure Among Adult Cancer Survivors

Development of heart failure (HF) among cancer survivors has become a major concern due to improvement in cancer treatments, shared risk factors, and disease mechanisms. HF risk varies by cancer type, and chemotherapy. Understanding these risks is crucial for effective cardiovascular monitoring. Florido et al. examined cancer-related HF risk in the ARIC study, adjusting for comorbidities, such as age, sex, smoking, BMI, lipid levels, hypertension, diabetes, and eGFR. They found that cancer survivors had a significantly higher risk of cardiovascular disease compared to those without cancer. Specifically, breast, lung, colorectal, and hematologic/lymphatic cancers were each significantly associated with increased risk, whereas prostate cancer was not [2]. Additional adjustments were made for hormone use in breast cancer (Table 1).

**Table 1:** Risk of Heart Failure among Cancer Survivors

Cancer Type	Hazard Ratio	95% CI
Any Cancer	1.37	1.26–1.50
Breast Cancer	1.32	1.08–1.60
Prostate Cancer	1.10	0.91–1.32
Lung Cancer	2.37	1.84–3.06
Colorectal Cancer	1.46	1.15–1.85
Hematopoietic and Lymphatic Cancer	2.70	2.04–3.59

### Heart Failure Among Survivors of Childhood Cancer

**Fig. 1:**
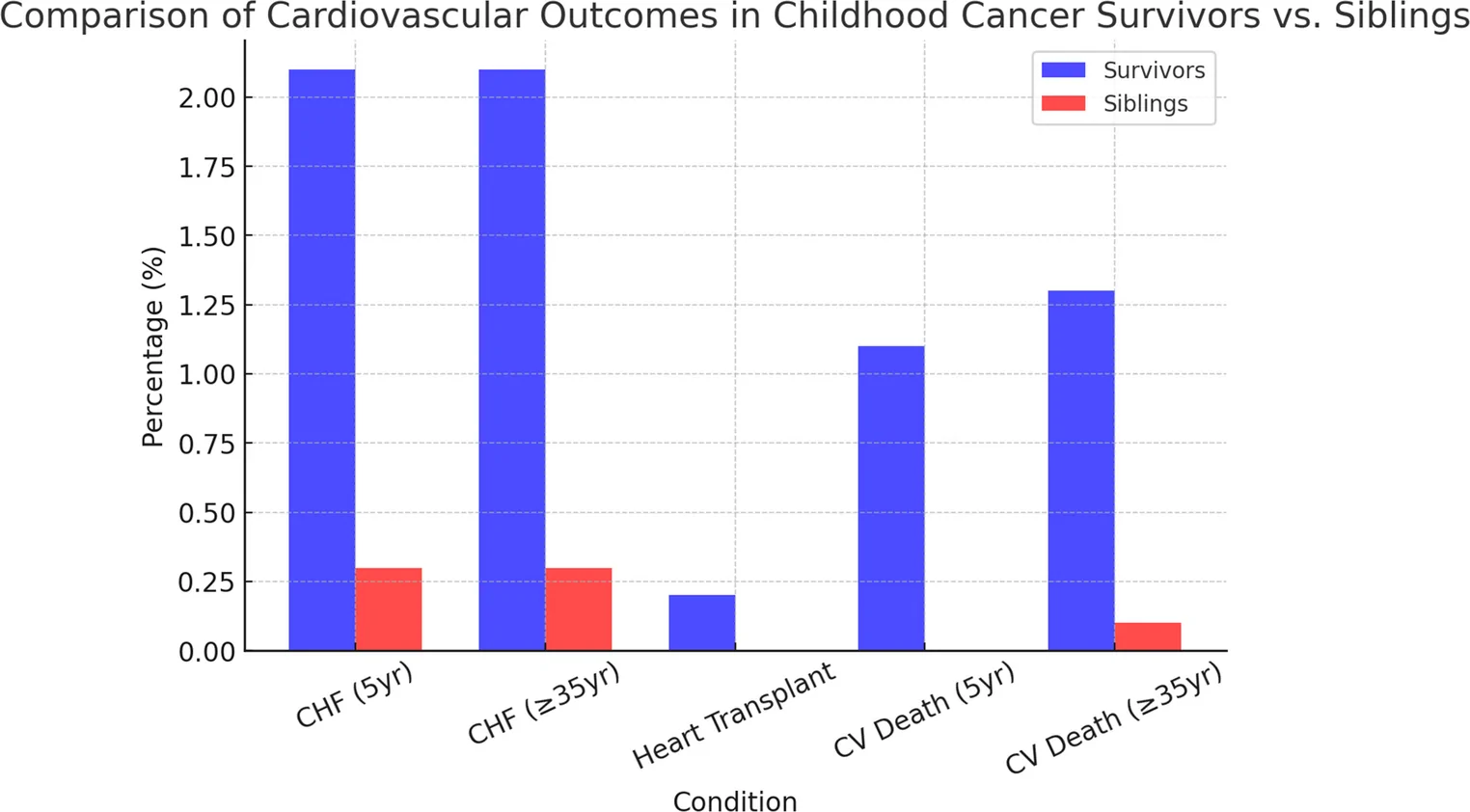
Prevalence of Heart Failure, Heart Transplantation and Cardiovascular Death between Siblings and Childhood Cancer Survivors. The blue graph demonstrates the risk among cancer survivors while the red graph demonstrates the risk among siblings with no cancer diagnosis The incidence of heart failure is 2.1% for cancer survivors as compared to 0.3% of siblings. The incidence of heart transplantation is 0.2% of survivors. The risk for cardiovascular mortality among cancer survivors was 1.1% and increased to 1.3% after age of 35

The risk of heart failure (HF) in childhood cancer increases significantly with age, particularly beyond the fourth decade of life, necessitating ongoing surveillance. Findings from the Childhood Cancer Survivor Study (CCSS), which followed over 7,600 survivors and siblings older than 35 years, highlight the elevated long-term HF risk in this population.[11, 12] Survivors encounter a 5-fold increased risk of stroke or myocardial infarction compared to healthy siblings with hypertension, dyslipidemia, diabetes, and obesity further increasing their cardiovascular risk [13]. (Fig. 1) Data suggests that hypertension alone significantly increases the risk of HF, particularly in those exposed to chest-directed radiotherapy (RT) and anthracyclines [14]. Despite advances in treatment, the burden of HF remains high. CCSS data suggests that 2.1% of survivors developed HF requiring medication by the age of 35, compared to 0.3% of siblings. Moreover, 0.2% of survivors required heart transplantation. Cardiovascular-related mortality was also higher, affecting 1.1% of survivors, and 1.3% after age 35 [13]. Among survivors aged 35 years and older, the hazard ratio for developing HF is 5.0 (95% CI), even after adjusting for age, race, and sex [13]. The Dutch Childhood Oncology Group-Long-Term Effects After Childhood Cancer (DCOG-LATER) study demonstrated that childhood cancer survivors treated with mitoxantrone, cyclophosphamide, anthracyclines, or radiotherapy had a 4.4% cumulative incidence of developing heart failure after median follow-up of 19.8 years [15].

## Patient, Cancer, And Treatment-Related Risk Factors for Heart Failure

### Patient-Related and Cancer-Related Risk Factors

Traditionally recognized risk factors for heart failure in combination with other cardiotoxic cancer treatment may further elevate the risk of heart failure. These pre-existing traditional risk factors can also exacerbate the risk of developing cancer treatment related cardiotoxicity thereby exponentially increasing the predisposition for developing heart failure. The underlying cancer diagnosis increases risk for heart failure in varying degrees as seen in Table 1.

### Cancer Therapies with Potential for Direct Cardiotoxicity

Anthracyclines cardiotoxicity is proposed to be brought about by oxidative stress leading to direct damage to cardiac myocytes, alteration of cell death pathways, and inhibition of topoisomerase-2 in cardiomyocytes.[16, 17] The incidence of development of cardiomyopathy following anthracycline therapy ranges from 3% to 48%, depending on the specific agent used and dose received [18]. The most predictive measure of developing cardiotoxicity is total cumulative dose of treatment. The incidence of early doxorubicin-induced heart failure was 3% with a cumulative dose of 400 mg/m2, 7% at a cumulative dose of 550mg/m[2] and 18% at a cumulative dose of 700mg/m^2 ^[19]. In addition, the concomitant use of other chemotherapeutic agents has also been shown to increase the risk of heart failure.

The mechanisms underlying HER2 therapy–related cardiotoxicity remain incompletely defined. Two proposed pathways include: [1] inhibition of HER2 (ErbB) signaling in cardiomyocytes, which disrupts critical cell survival and stress response pathways, leading to impaired contractility, and [2] eNOS downregulation leading to mitochondrial dysfunction and oxidative stress [20]. For HER-2 targeted chemotherapeutic agents such as trastuzumab, the dose of treatment has not been associated with increased cardiotoxic effects but is related to the duration of therapy. Patients who received longer duration of therapy with trastuzumab had additional cardiotoxicity compared to those with shorter therapy [21]. In addition, prior history of radiotherapy and exposure to anthracyclines have been identified as risk factors for HER-2 therapy cardiotoxicity [22].

### Cancer Therapies Associated with Secondary Cardiovascular Complication

Immune checkpoint inhibitors (ICIs) are associated with a spectrum of immune-mediated cardiovascular adverse events, including myocarditis, pericarditis, arrhythmias, pericardial effusion, vasculitis, accelerated atherosclerosis, heart failure, and acute coronary syndromes [23]. Myocarditis is the most recognized and potentially fatal complication, with mortality up to 60% [23]. Heart failure is a recognized complication of ICI therapy and may arise through both inflammatory mechanisms, secondary to myocarditis, or through non-inflammatory mechanisms [24]. Patients have a higher risk for ICI cardiotoxicity when they have undergone or are undergoing dual ICI therapy, a combination of ICI with other cardiotoxic therapies, and those with previous cardiovascular risk factors and/or disease before initiation of ICI therapy.

Chimeric antigen receptor T-cell therapy (CAR-T) frequently induces cytokine release syndrome (CRS), which can precipitate arrhythmias, hypotension, myocardial depression, cardiomyopathy, and heart failure [25]. The reported incidence of cardiomyopathy and heart failure after CAR-T therapy is estimated to be from 1–15% [26].

Radiation therapy, particularly when involving the thorax, is linked to both macrovascular and microvascular injury, manifesting as accelerated coronary atherosclerosis, myocardial ischemia, fibrosis, pericarditis, valvular disease, conduction disturbances, and autonomic dysfunction [27]. Coronary artery disease is the most common adverse effect with an incidence of up to 85% [28]. The prevalence of cardiomyopathy following radiation therapy is estimated to be up to 10% [27, 28]. The main determinants of radiation therapy-associated cardiovascular disease include pre-existing risk factors, the dose of radiotherapy to cardiovascular structures, age at the time of exposure, the time elapsed since exposure, and concurrent use of other chemotherapeutic agents [29]. Multiple studies have shown that a linear dose-dependent relationship exists between the radiation received and the risk of cardiotoxicity, and no safe dose has been identified [30, 31].

### Genetic and Epigenetic Factors

It has been hypothesized that even after accounting for known risk factors such as known preexisting cardiac risk factors, age, sex, and the dosage and length of certain cancer therapies, some patients are still more susceptible to developing cardiotoxicity from cancer therapy.

Titin-truncating variants (TTNtvs) occur in 7.5% of patients who developed cancer-therapy induced cardiomyopathy compared to 0.6% of the reference population [32]. Prior studies have proposed that evaluation of the patients’ genotype improves the ability to identify patients who may be at higher risk for these adverse cardiac effects [32].

In addition, multiple studies have looked specifically at genes associated with anthracycline-induced cardiotoxicity and some 40 candidate genes and single nucleotide polymorphisms have been identified [30]. Studies on genetic variants in doxorubicin transport and free radical metabolism have identified 5 significant associations with polymorphisms of the NAD(P)H oxidase and doxorubicin efflux transporters [33]. Blanco et al. found that individuals with homozygous G genotypes of CBR3 V244M, exposure to low to moderate doses of anthracyclines increased cardiomyopathy risk when compared with individuals with non-homozygous G variants [34]. Currently, the routine use of genetic testing to evaluate cardiotoxicity risk prior to initiation of cancer therapy is not recommended; however, with more findings of these genetic risk factors, in the future, a targeted intervention may be helpful for patients at increased risk of cardiomyopathy [30].

##  Cardiac and Heart Failure Risk Calculators among Patients with Cancer

Risk prediction models are instrumental in determining individualized frequency of follow-up and testing for this at-risk population. Optimal risk prediction models should be practical, accurate, and generalizable. These models should utilize variables commonly available in electronic medical records and should be easily implemented. Accuracy of these risk calculators allows for the determination of patients who will develop the disease (i.e. heart failure) and consistent predictions with observed outcomes. Generalizability allows for the risk model to be used across different patient populations particularly among women and racial minorities [35].

Several risk calculators and predictive models have been tested and validated to determine risk of major cardiovascular events (MACE) including myocardial infarction, heart failure, arrhythmia and transient ischemic attack or stroke among patients undergoing cancer therapy and cancer survivors.

Heart failure risk among cancer patients is assessed before, during, and after cancer treatment. The goal of assessing HF risk before treatment is to identify high risk patients, provide guidance on risk factor modification, and how to tailor cancer therapy to minimize further heart failure risk. During cancer treatment, the goal is for early detection of heart failure and determining frequency and timing of monitoring with biomarkers, imaging studies, symptoms, and clinical exam. After cancer treatment, the goal for cancer survivors is for continued monitoring and risk factor modification. Heart failure risk persists even decades after treatment and risk increases with aging and comorbidities highlighting the importance of survivorship care.

### General Population Cardiovascular and Heart Failure Risk Calculators

The American Heart Association has released a novel sex-specific, race-free model that predicts 10-year and 30-year risk of total cardiovascular disease (including atherosclerotic cardiovascular disease and heart failure) among adults. The PREVENT equation incorporates cardio-kidney-metabolic health in determining risk. It provides an updated version of the ACC/AHA ASCVD risk calculator [36]. The ARIC HF risk score adds Nt pro BNP to the ARIC risk scores and provides a validated 10-year risk of incident HF[37]. The PCP HF calculator provides a validated 5-year risk prediction of HF regardless of sex and race [38]. These calculators should ideally be utilized prior to cancer therapy initiation as it accounts for baseline CVD risk assessment. However, these calculators do not account for cancer as a risk factor which already limits their utility since a new cancer diagnosis increases the risk for heart failure with a HR of 1.62 [10].

### Cancer Specific Cardiovascular Risk Calculators

There are several cancer specific risk calculators that have been validated to determine risk of cardiovascular events at the time of diagnosis and treatment. The HFA ICOS (Heart Failure Association and the International Cardio-Oncology Society) risk score was noted to be more sensitive than SCORE (Systematic Coronary Risk Evaluation) chart in identifying risk of cardiovascular toxicity in patients with CML (*p*< 0.01) [39]. The CHEMO-RADIAT score is based on both conventional and cardiovascular risk factors and breast cancer treatment related risk factors in predicting MACE among breast cancer survivors with time dependent C indices at 3 years and 7 years of 0.87 and 0.842, respectively, in the validation cohort [40]. The CARE-BMT score (Cardiovascular Registry in Bone Marrow Transplantation) is a point-based risk score that classifies patients undergoing pre-hematopoietic stem cell transplantation (HSCT) as low-, intermediate-, and high-risk with the 5-year cumulative incidence of cardiovascular events being 4%, 10.3%, and 22.4%, respectively. The AUC for predicting cardiovascular events at 100 days, 5 and 10 years post HSCT were 0.65, 0.73, and 0.76 respectively [41].

Among long-term survivors of childhood cancer, The Childhood and Adolescence Cancer Survivor CardioVascular (CHACS-CV) score has been validated in a large dataset from the National Cancer Institute. The risk score uses 8 variables to provide cardiovascular risk stratification with the goal of identifying those at increased cardiovascular risk and in need of closer follow-up and early intervention with risk-reduction strategies. This risk score showed good discrimination for CV mortality with a Harrell’s C-index of 0.73 [0.68–0.78], *p*< 0.001 [42]. These risk calculators assess general cardiovascular risk among patients with cancer.

### Heart Failure Specific Risk Scores

Heart failure specific risk scores generally identify risk of cancer therapy related cardiac dysfunction at the time of cancer therapy or shortly thereafter. (Table 2) There is limited data on available risk scores that identify heart failure risk in long term cancer survivors but recent publications, such as CHERISH, have aimed to address this gap [43]. Existing risk factors often identify CTRCD or HFrEF but there is limited data for identifying risk for heart failure with preserved ejection fraction.

**Table 2:** HF Risk Calculators for Patients with Cancer

Risk Calculator	Variables Included	Study Population	Condition of Interest	Risk stratification	Outcome	Validation
Lyon et al. (HFA ICOS) [64]	Previous cardiovascular disease, cardiac biomarkers, demographics, co morbid conditions, previous cardiotoxic cancer treatment, smoking, BMI	Patients with cancer who are about to receive cancer therapy	Patients receiving anthracycline, HER 2 targeted therapy, VEGF inhibitors, second and third generation TK, multiple myeloma therapies (proteasome Inhibitors and immunomodulatory drugs), RAF and MEK inhibitors, androgen deprivation therapies	Stratify patients into low, medium, high, and very high risk for cardiovascular complications	Stratified patients into different risk categories. The ESC HFA ICOS statement used the risk stratification to determine frequency of biomarker and imaging testing.	Not validated at the time of publication but have been validated by other studies with noted heterogeneity in results depending on type of cancer and type of cancer therapy.
Liu et al. (ABSDELL model) [45]	Age, BMI, SBP, DBP, E-wave, LVESD, LVEF	Patients with HER2 positive breast cancer and treated with chemotherapy and trastuzumab (*n* = 212)	Patients with HER 2 positive breast cancer	Variables are included in an online risk calculator that provides a graph of LVEF decline risk, 95% decline probability and a numerical summary	Quantitatively predict the risk of LVEF decline over 3 years in breast cancer patients treated with trastuzumab	AUC was 0.802 in 1 year and 0.881 in 3 years. Internal validation c-index was 0.801 in 1 year and 0.881 in 3 years.
Chow et al. (CCSS-CHF) [53]	Age, sex, anthracycline and chest radiotherapy dose	Childhood survivors of cancer with no evidence of cardiovascular disease 5 years after cancer diagnosis (*n* = 13,060)	Survivors of any childhood cancer with prior anthracycline and radiotherapy treatment	Classify patients to low-, moderate-, high-risk groups according to cumulative incidence of heart failure at age 40 years of 0.5%, 2.4%, and 11.7% respectively.	Predict individual HF risk through age 40 years	Included international cohorts – Emma Children’s Hospital, National Wilms Tumor Study, and St Jude Lifetime Cohort Study to validate CCSS prediction model.
Herrmann et al. (Mayo Clinic Cardiotoxicity Risk Score) [51]	Type of cancer chemotherapy, sex, age, concurrent chest radiation, diabetes mellitus, HTN, CAD, known HF	Patients who are about to undergo chemotherapy.	All cancer patients undergoing chemotherapy	Very low (0 points), Low (1–2 points), Intermediate (3–4 points), High (5–6 points), Very high > 6 points)	Detect CV risk among patients with prior cancer therapy.	Not validated at the time of publication but has been used in other studies that demonstrate that it predicts cardiac events with a HR of 2.664
Ezaz et al.[50] (Risk Prediction Model for Heart Failure and Cardiomyopathy After Adjuvant Trastuzumab Therapy for Breast Cancer)	Age, adjuvant chemotherapy, coronary artery disease, atrial fibrillation or flutter, diabetes mellitus, hypertension, renal failure	SEER Medicare database identified women with breast cancer who received adjuvant trastuzumab (*n* = 1664)	Patients with HER 2 positive breast cancer	Low risk (0 to 3 points), Medium (4 to 5 points), High (*≥* 6 points)	Risk score stratified 3-year risk of HF between lowest and highest risk groups by 2-fold	Validation cohort showed that 3 year HF risk increased as risk score increased with noted *p* < 0.001.
NSABP-31 CRS [65]	Age, LVEF, Use of trastuzumab, anthracyclines	Women with HER2 positive breast cancer who received doxorubicin and cyclophosphamide followed by paclitaxel vs. paclitaxel and trastuzumab (*n* = 944)	Patients with HER 2 Positive Breast Cancer	Calculated a cardiac risk score according to: $$\frac{7.0 + (0.04 \times \\\text{Age in years}) \\- (0.1 \times \\\text{Baseline percent LVEF})}{4.76}$$ And plotted against the probability of a cardiac event	Identify probability of HF and associated with decrease in LVEF of > 10% points to a value less than 55% or a decrease of more than 5% to a value below the lower limit of normal	Validated with HFA ICOS and noted AUC of 0.51–0.60 in discriminating for CTRCD
Yu et al.[44] (Nomogram for Predicting Risk of CTRCD in Patients with HER2 + Breast Cancer)	Age, race, BMI, LVEF, SBP, CAD, Diabetes Mellitus, Arrhythmia, Anthracycline Exposure	Consecutive patients with HER2 positive breast cancer treated with trastuzumab therapy (*n* = 1440)	Patients with HER-2 breast cancer	Points were assigned to each of the variables and placed on a normogram	Predicts 1-year risk of CTRCD during HER2 targeted breast cancer therapy	Validated for calibration and discrimination using bootstrap resampling. Concordance index noted to be 0.687.
Soh et al. (CHERISH) [43]	Age, years since cancer diagnosis, CAD, arrhythmia, MI, DM, HTN, HLD, leukemia, non-Hodkin lymphoma, lung cancer, and breast cancer.	Cancer survivors with prior leukemia, non-Hodgkin lymphoma, lung cancer, and breast cancer.	Cancer survivors	Determine 10-year HF incidence	Integrating CHERISH score into EMR may provide large scale automated HF risk assessment among cancer survivors.	Predicted 10-year HF incidence with AUC of 0.829 during internal validation and AUC of 0.721 during external validation.
Barac et al.[52] (Risk Prediction Model for Development of Heart Failure or Cardiomyopathy After Breast Cancer Treatment)	Age, race, ethnicity, socioeconomic status, breast cancer treatment, cancer stage, obesity, HTN, DM, HLD, smoking	Women with newly diagnosed invasive local or regional breast cancer (*N* = 26044)	Early breast cancer patients	Risk of heart failure or cardiomyopathy at 10 years	Identified low and high risk patients at risk of heart failure and cardiomyopathy at 10 years with an	AUC of 0.79 at 10 years in the validation cohort.

A risk nomogram based on 9 readily available variables has been created by Yu et al. with the goal of predicting 1-year risk of cancer therapy related cardiac dysfunction (CTRCD) during human epidermal growth factor receptor 2-targeted breast cancer therapy. The nomogram allocated points for several variables, and the total number of points determined the probability of CTRCD. Results demonstrated good discrimination and model calibration [44]. The ABSDELL model (Age, BMI, SBP, DBP, E-wave, LVESD and LVEF) quantitatively predicts the risk of LVEF declining over 3 years in breast cancer patients on trastuzumab. It has both a nomogram and an online calculator available for clinician use and has shown good validity [45].

HFA ICOS and Atherosclerosis Risk in Communities (ARIC HF) outperformed the Pooled Cohort Equations to Prevent Heart Failure (PCP HF) risk score in predicting incident HF among patients with breast cancer and lymphoma; however, none of these risk scores demonstrated discriminative ability that is significantly superior [46]. Validation of the HFA ICOS risk score for other cancers have also shown that its use is limited among patients with BRAF and MEK inhibitor associated CTRCD [47]. Comparison of the National Surgical Adjuvant Breast and Bowel Project B-31 cardiac risk score (NSABP-31 CRS) and HFA-ICOS demonstrated ability to identify absolute risk of CTRCD only among the highest risk patients in women with HER2-positive breast cancer [48]. Another validation study on HFA-ICOS risk score also showed that it is most predictive of cardiotoxicity only in very-high-risk patients and is less predictive in lower risk cohorts of patients with HER2-positive breast cancer [49]. Ezaz et al. also developed a risk score that stratified the 3 year risk of heart failure among patients with HER 2 positive breast cancer who have received adjuvant chemotherapy [50]. The Mayo Cardiac Risk Score similar to the HFA ICOS score provides a general overview on risk of cardiovascular events including heart failure across all patients who have received cancer treatment [51]. The cancer-specific HF prediction from EMRs in survivor health care (CHERISH) risk score was developed from the UK Biobank and has been validated in a population-based Ontario cohort. It demonstrated a strong prediction of 10-year HF incidence among cancer survivors when compared to ARIC-HF (AUC 0.829 vs. 0.697) [43]. A recent publication on a risk-model that identified 10-year risk of heart failure or cardiomyopathy among early stage breast cancer patients showed good calibration and high accuracy even on the validation cohort [52].

Compared to survivors of adult cancers, survivors of childhood cancers have an externally validated risk calculator to determine risk of HF. The Childhood Cancer Survivor Study (CCSS) utilizes several variables including sex, age at cancer diagnosis, anthracycline and chest radiotherapy dose to determine the corresponding risk of heart failure, ischemic heart disease, and stroke. The online calculator provides the percentage 10-year risk and risk at age 50 for each of these cardiac outcomes [53].

### Limitations of Current Heart Failure Risk Prediction Models

The heterogeneity of cancer treatment and cancer diagnosis makes it challenging to individualize HF risk among cancer survivors. Existing models also often focus on cancer patients treated with medications that can cause CTRCD so most risk calculators focus on breast cancer and lymphoma patients. Current studies have data assessing risk within the first few years of cancer treatment; however, there is a lack of long-term studies assessing lifetime risk for cancer survivors. The study by Barac et al. only extends to predicting risk for HF from 10 years after treatment completion [52]. The variability in how patients are treated also makes it more challenging to develop a risk stratification tool that can encompass all these factors. Heart failure is also often assessed in terms of CTRCD and cardiomyopathy with limited data on heart failure with preserved ejection fraction (HFpEF). HFpEF is equally relevant in this population due to the elevated risk of cardio-kidney-metabolic syndrome given the shared risk factors for HF and cancer. Radiation therapy, immunotherapy, and other cancer therapies can lead to coronary artery disease, restrictive, and constrictive cardiomyopathy that all increase the risk for HFpEF. Among cancer survivors, HFpEF is seen frequently in several published studies but larger data and longer follow up are needed to better assess the burden of disease. The need for more studies and data on HFpEF in this population is highlighted in the ESC Scientific Statement on HFpEF in cancer patients and survivors [54].

## Managing Patients with Elevated Risk for Heart Failure

Diagnosing heart failure among patients with cancer remains a clinical challenge due to significant symptom overlap—fatigue, dyspnea, and peripheral edema, which are seen in both cancer progression in terminal patients and heart failure. Lack of standardized definition of HF as a complication from chemotherapy versus clinical deterioration from cancer disease burden further limits diagnostic clarity.

Given these diagnostic challenges, it is crucial to establish comprehensive baseline cardiovascular assessment which will help with risk stratification and guide surveillance planning throughout cancer therapy. Utilizing available risk stratification tools (i.e. PREVENT) helps identify pre-existing cardiovascular risk and tailor monitoring and follow-up and facilitate shared decision-making to support individualized management.

The HFSA scientific statement on cardio-oncology and heart failure emphasizes the importance of multidisciplinary care and structured surveillance. The scientific statement has addressed key clinical questions related to oncology and heart failure. However, there are some questions that persist including the use of cardiac biomarkers such as troponin and natriuretic peptides, direction on long-term post-treatment cardiac surveillance, monitoring protocols for patients receiving therapies such as immune checkpoint inhibitors, proteasome inhibitors, and CAR T-cell therapy and guidance on risk stratification or treatment modification in patients with pre-existing cardiovascular disease or high-risk clinical features. Absence of granularity in management protocol poses difficulties in managing patients with elevated baseline cardiovascular risk. Moreover, there is limited evidence on clinical decisions for dose adjustments of cardiotoxic therapies or the use of adjunctive cardioprotective agents such as beta-blockers, ACE inhibitors, MRA, SGLT2i, and statins in elevated based line heart failure risk [3].

The 2022 ESC/ICOS cardio-oncology guidelines offer an algorithm-based approach to evaluating cardiovascular risk and monitoring for cardiac toxicity based on the type of cancer treatment [30]. Similar to the HFSA statement, the role of biomarkers, multimodality imaging are highlighted for early detection of cardiovascular disease. As compared to the HFSA statement, the 2022 ESC/ICOS provides a more general cardiovascular risk overview as compared to focusing on cardiac dysfunction but has more structured flowcharts for clinician use. Table 3 provides a comparison between the two guidelines.

**Table 3:** Comparison between HFSA and ESC/ICOS Statements on Managing Cancer Patients and Heart Failure

	HFSA 2025 Statement	ESC/ICOS 2022 Statement
Risk Prediction and Stratification	Recommending evaluation or risk: pre- during, and post- cancer treatment No specific risk stratification tool recommended Incorporating traditional HF risk factors (HTN, DM, Obesity) with novel contributors (CHIP)	Recommending evaluation of risk: pre-, during, and post- cancer treatment and the use of the HFA-ICOS risk stratification tool Use of biomarkers and imaging and incorporating therapy specific risk during assessment
Diagnosis of Heart Failure	Utilizes the ACC/AHA staging A-D and classification according to ejection fraction	Defined CTRCD as symptomatic vs. asymptomatic and classified it according to severity (mild, moderate, severe)
Management Heart Failure	Emphasized the role of starting GDMT when able	Recommended starting beta blockers and ACEi/ARB/ARNI with LVEF decline Provides flowcharts and algorithms according treatment therapy and risk
Monitoring for Heart Failure	Recommends continuous surveillance with frequency determined by baseline risk and type of cancer therapy. No specific intervals mentioned but highlights tailoring patient specific plans and the future role of AI	Provides structured timelines and surveillance testing at 3-, 6-, 12-month intervals and provides specific 2-, 3- and 5- year plan for adult and childhood survivors of cancer

The 2022 ESC Cardio-oncology guidelines have provided guidance on the frequency of imaging and CV biomarker monitoring depending on the type of cancer treatment (Table 4) [30]. They recommend that anthracyclines or HER-2 targeted therapies should not be administered to patients LVEF < 40% unless there is no effective alternative cancer therapy. For patients with moderately reduced LVEF (40–49%) or those with normal LVEF who are at elevated risk due to cumulative exposure to cardiotoxic agents and underlying cardiovascular comorbidities, anthracyclines or HER-2 inhibitors may be considered in conjunction with cardioprotective strategies which include the prophylactic use of angiotensin-converting enzyme inhibitors (ACEIs) or angiotensin receptor blockers (ARBs), and/or beta-blockers, to decrease the risk of anthracycline induced cardiomyopathy and to preserve myocardial function during treatment. All patients receiving HER2 inhibitors should undergo comprehensive cardio-oncologic evaluation, including imaging, every 3 months throughout treatment [55]. Recommendations and real-world clinical practice on the utility of troponin in predicting left ventricular systolic dysfunction (LVSD) during therapy are variable due to inconsistent findings across studies [56–60]. However, the 2020 joint guideline writing group supports its use in clinical scenarios with the strongest supporting evidence—such as high cumulative-dose anthracyclines (doxorubicin ≥ 250 mg/m² or epirubicin ≥ 600 mg/m²), lower doses given in combination with other cardiotoxic agents, or in the presence of underlying cardiovascular risk factors—provided that serial measurements are performed by the same laboratory using consistent assay methods and timing (within 24 h post-chemo infusion). The utility of troponin in predicting cardiotoxicity from HER2-targeted inhibitors remains inconclusive overall but may offer greater predictive value in patients with prior anthracycline exposure, and its use after each infusion may be considered in high-risk individuals [61].

**Table 4:** ESC 2022 Guidelines on Surveillance for Cardiotoxicity during Cancer Therapy [30]

Therapy	Baseline Monitoring	During Treatment	Post-Treatment Surveillance
**Anthracyclines**	EKG Trop/BNP TTE (LVEF, GLS)	Cardiac enzymes every other chemo cycle for low and moderate risk and every cycle for high risk TTE every 100 mg/m² after 200–250 mg/m²(doxorubicin)for low and moderate risk and every other chemo cycle for high risk	Cardiac enzymes at 3 months for everyone and at 1 year for high risk. TTE at completion, for 3 months, 1 year, then individualized based on risk (high-risk: at 1 & 5 years)
**HER2-Targeted Therapy**	EKG Cardiac enzyme TTE	Cardiac enzymes every 3 months TTE every 3 months	TTE at 3 months and then every 6 months − 1 year for 2 years in high risk.
**VEGFi** **(monoclonal Ab and TKI)**	EKG and TTE in low risk EKG, cardiac enzymes and echo in moderate to high risk	Cardiac enzymes every 4 months for moderate risk and every 3 months for high risk TTE every 4 months for moderate risk and every 3 months for high risk	Individualized surveillance
**Radiation Therapy (RT)**	TTE if high-risk	Not required unless symptomatic	Annual physical Exam and EKG TTE at 10 years (for low risk) or at 5 years (for high risk), then every 5 years Stress test at the time of onset of exertional symptoms
**BCR-ABL TKI**	EKG TTE	Clinical monitoring, repeat echo for high-risk patients	Individualized surveillance
**Proteasome Inhibitors (PIs)**	EKG Cardiac enzymes TTE (LVEF)	Cardiac enzyme at every cycle for first 6 cycles TTE at every 3 cycles.	Individualized approach
**BRAF/MEK Inhibitors**	EKG TTE (LVEF)	EKG at 2 and 4 week then every 3 months TTE at every 4 months in moderate and high risk	Individualized approach
**Immune Checkpoint Inhibitors (ICIs)**	EKG TTE, Troponin, BNP	EKG every cycle up to 4th cycle then every 6–12 month Cardiac enzymes every cycle up to 4th cycle then every 6–12 month TTE if clinically indicated	Not required unless symptoms develop
**CAR T-Cell Therapy**	EKG Cardiac enzymes TTE	Repeat imaging if severe cytokine release syndrome occurs	Not well-defined; assess case-by-case
**Hematopoietic Stem cell transplant**	EKG Cardiac enzymes TTE CPET (in selected patients)		EKG and Cardiac enzymes at every 3 months up to 1 year then yearly TTE at every 3 months only in high- risk individual

With the available risk prediction models and cardio oncology guidelines, clinicians are now at the crossroads of what to do with all this data and how to incorporate them in clinical care. When available, cancer therapy specific risk calculators are preferred as they provide more nuanced data and prognostication as seen in Table 2. The HFA/ICOS risk calculator can be used for cancer patients whose treatment do not have specific risk calculators. Once risk is identified, clinicians and patients will discuss need for further testing (biomarkers and/or imaging studies) and how to modify long term risk. Guidance on lifestyle modification is also important to be conveyed in this part of cardio oncology management. Heart failure management should incorporate HFSA recommendations regarding GDMT initiation and surveillance. Patients should be informed of the lack of long-term risk assessment tools for heart failure post survivorship; however, they remain at elevated risk for developing HFpEF due to the elevated risk from the cancer itself and cancer treatment [54].

It is important to acknowledge the cardio oncology expertise is not widely available and is often limited to specialized centers. Lack of standardized referral pathways, resources, and invested stakeholders all limit successful use of these risk prediction models that will translate to clinical action. Strategies such as embedding risk algorithms into electronic medical records similar to what was done in CHERISH can help easier identification of patients who need further diagnostic testing and surveillance visits. Ultimately, periodic reevaluation of these interventions are needed as strategies have to be tailored to the institution’s resources and to the patient’s needs [62].

## Future Directions

Future studies that analyze large cohorts of data may provide better heart failure risk stratification models by integrating more variables that have not been previously used in published risk calculators. Studies that also have longer follow-up may provide better lifetime risk assessment among cancer survivors. The use of machine learning driven models can provide more personalized risk assessment and more precise recommendations after accounting for the patient’s baseline risk factors, cancer type, and concomitant cancer specific therapy [63]. Guidance on long term monitoring among the survivorship population is also an area that needs to be further explored. Frequency of cardiac imaging, biomarker testing, clinical assessment, and timing of referral to cardio-oncology clinics based on prior cancer therapy regimen and underlying risk factors remain to be determined. As highlighted in this review article, this calls for stronger collaboration between oncologists and cardiologists to allow for disease prevention and timely management.

## Conclusion

With the advancement of diagnosis and treatment options, cancer patients have improved survival. HF risk assessment should be done before, during, and after cancer treatment because a cancer patient’s risk for HF is dynamic. With the improvement in mortality, cancer survivors are at an elevated risk of cardiovascular disease particularly heart failure due to baseline risk factors, cancer diagnosis, and prior cancer therapy. Current CVD and HF risk calculators are predominantly used pre- and during- cancer treatment and often incorporate a generalized approach without accounting for the specific type of cancer and therapy. There is a gap in prediction models that assess lifetime HF risk and guidance on long term follow up among cancer survivors. This article highlights the current evidence, and the challenges associated with predicting heart failure in the growing population of cancer survivors.

## Key References

- Lyon AR, López-Fernández T, Couch LS, et al. 2022 ESC Guidelines on cardio-oncology developed in collaboration with the European Hematology Association (EHA), the European Society for Therapeutic Radiology and Oncology (ESTRO) and the International Cardio-Oncology Society (IC-OS). Eur Heart J. 2022;43(41):4229-4361.
  - ○ This paper is the first cardio-oncology guideline that outlines risk stratification, surveillance, and management for patients with cancer and for cancer survivors.
- Keramida K, Lopez-Fernandez T, Anker MS, Petrie MC, Ameri P, Anderson LJ, Bergler-Klein J, Deswal A, Farmakis D, Ibañez B, Lam CSP, Lang NN, Lyon AR, Mirabel M, Moura B, Paulus WJ, Rakisheva A, Savarese G, Senni M, Tocchetti CG, Metra M, Filippatos G. Heart failure with preserved ejection fraction in cancer patients and survivors. A scientific statement of the Heart Failure Association of the ESC and the ESC Council of Cardio-Oncology. Eur J Heart Fail. 2025 Nov;27(11):2152-2167. doi: 10.1002/ejhf.70005. Epub 2025 Sep 12.
  - ○ This is the most recent scientific statement that specifically focuses on heart failure with preserved ejection fraction among cancer patients and survivors.
- Sen J, Amir E, Austin PC, Marwick TH, McIntosh C, Thavendiranathan P, Abdel-Qadir H. Risk Prediction in Cardio-Oncology: Conceptual and Methodological Considerations: JACC: CardioOncology State-of-the-Art Review. JACC CardioOncol. 2026 Apr;8(2):101-119. doi: 10.1016/j.jaccao.2026.01.006.
  - ○ A recent review that summarizes the latest data on risk prediction models in cardio oncology.

## Main Messages

- With the improvement in cancer therapy, there is a growing number of cancer patients who have higher long-term risk of heart failure.
- Challenges exist in diagnosing and assessing HF in the cancer population and current heart failure risk scores often do not account for cancer-related heart failure risk.
- Most risk prediction models focus on assessing risk before and during cancer treatment but there is a lack of risk calculators for cancer survivors.

## Data Availability

No datasets were generated or analysed during the current study.

## Abbreviations

ACEIs: Angiotensin-converting enzyme inhibitors
ARBs: Angiotensin receptor blockers
AIC: Anthracycline-induced cardiomyopathy
ARIC HF: Atherosclerosis Risk in Communities
CTRCD: Cancer therapy related cardiac dysfunction
CVD: Cardiovascular disease
CARE-BMT: Cardiovascular Registry in Bone Marrow Transplantation
CHACS-CV: Childhood and Adolescence Cancer Survivor CardioVascular
CCSS: Childhood Cancer Survivor Study
CAR: Chimeric antigen receptor
CAR-T: Chimeric antigen receptor-modified T cells
CRS: Cytokine release syndrome
DCOG-LATER: Dutch Childhood Oncology Group-Long-Term Effects After Childhood Cancer study
GDMT: Guideline-directed medical therapy
HF: Heart failure
HFA ICOS: Heart Failure Association and the International Cardio-Oncology Society
HSCT: Hematopoietic stem cell transplantation
ICIs: Immune checkpoint inhibitors
LVSD: Left ventricular systolic dysfunction
MACE: Major cardiovascular events
NSABP-31 CRS: National Surgical Adjuvant Breast and Bowel Project B-31 cardiac risk score
PCP HF: Pooled Cohort Equations to Prevent Heart Failure
RT: Radiotherapy
SCORE: Systematic Coronary Risk Evaluation
TTNtvs: Titan-truncating variants

## Declarations

### Human or Animal

This article does not contain any studies with human or animal subjects performed by any of the authors.
